# Biofilm inhibitory effect of alginate lyases on mucoid *P. aeruginosa* from a cystic fibrosis patient

**DOI:** 10.1016/j.bbrep.2021.101028

**Published:** 2021-05-26

**Authors:** Sonal Mahajan, Sonali Sunsunwal, Vikas Gautam, Meenu Singh, T.N.C. Ramya

**Affiliations:** a CSIR- Institute of Microbial Technology, Sector 39A, Chandigarh, 160036, India; b Department of Medical Microbiology, Post Graduate Institute of Medical Education and Research, Sector 12, Chandigarh, 160012, India; c Advanced Pediatrics Centre, Post Graduate Institute of Medical Education and Research, Sector 12, Chandigarh, 160012, India

**Keywords:** *Pseudomonas aeruginosa*, Biofilm, Cystic fibrosis, Mucoid, Sputum, Alginate lyase, *Cellulophaga algicola*, *Ca*Aly, *Cellulophaga algicola* alginate lyase

## Abstract

Chronic mucoid *Pseudomonas aeruginosa* infections are a major scourge in cystic fibrosis patients. Mucoid *P. aeruginosa* displays structured alginate-rich biofilms that are resistant to antibiotics. Here, we have assessed the efficacy of a panel of alginate lyases in combating mucoid *P. aeruginosa* biofilms in cystic fibrosis. Albeit we could not demonstrate alginate degradation by alginate lyases in sputum, we demonstrate that the endotypic alginate lyases, *Ca*Aly (from *Cellulophaga algicola*) and *Vsp*AlyVI (from *Vibrio* sp. QY101) and the exotypic alginate lyases, *Fsp*AlyFRB (from *Falsirhodobacterium* sp. alg1), and SA1-IV (from *Sphingomonas* sp. A1), indeed inhibit biofilm formation by a mucoid *P. aeruginosa* strain isolated from the sputum of a cystic fibrosis patient with comparative effect to that of the glycoside hydrolase PslG, a promising candidate for biofilm treatment. We believe that these enzymes should be explored for in vivo efficacy in future studies.

## Highlights

• A *P. aeruginosa* strain was isolated from the sputum of a cystic fibrosis patient.
• The anti-biofilm efficacy of endotypic and exotypic alginate lyases was assessed.
• Alginate lyases *Ca*Aly, *Vsp*AlyVI, *Fsp*AlyFRB, and SA1-IV inhibited biofilm formation.
• Similar anti-biofilm effect was observed for the glycoside hydrolase, PslG.

## 1 Introduction

Cystic fibrosis patients are prone to lung infections by opportunistic pathogens, primarily *Pseudomonas aeruginosa*. *P. aeruginosa* has refined mechanisms of adaptation (such as mucoid conversion and biofilm formation) to cystic fibrosis airway conditions (such as oxygen deficiency, immune system response and oxidative stress). Clinical mucoid isolates of *P. aeruginosa* from cystic fibrosis patients display alginate overexpression, resulting in structured biofilms with increased resistance to antibiotics, which is associated with heightened morbidity, and mortality [1]. Alginate is absent in human tissues and is a promising molecule to target. Therefore, alginate lyases (enzymes that break down alginate, endolytically or exolytically, into unsaturated products following a beta-elimination reaction) have been explored as biotherapeutics for cystic fibrosis [2,3] but it is questionable whether catalysis-dependent biofilm dispersion was observed in these studies [4]. Further, alginate lyases that are capable of degrading alginate in *Pseudomonas* biofilms have been reported to be inactivated in the presence of the high concentrations of Zn^2+^ and Ca^2+^ ions present in the secretions of cystic fibrosis patients [5]. Therefore, there is a need for further assessing the anti-biofilm potential of alginate lyases in clearing biofilms of *P. aeruginosa* in cystic fibrosis.

In this study, we have employed a panel of biochemically characterized, exolytic and endolytic alginate lyases from different sources. They include (1) endolytic alginate lyase, SA1-III from *Sphigomonas* sp. strain A1 (GenBank: BAB03312.1) which is optimally active at 70 °C and pH 8 and shows high efficacy against acetylated alginates from *Pseudomonas* sp. isolated from mucous samples of cystic fibrosis patients, (2) endolytic alginate lyase, PspAlgL from *Pseudomonas* sp. QD03 (GenBank AAR23929.1), which shows optimal activity at 37 °C and pH 7.5 against polyM and acetylated alginate from *Pseudomonas aeruginosa* FRD1 with enhancement of activity in the presence of Ca^2+^ and no inhibition effect by Zn^2+^, (3) exolytic alginate lyase, SA1-IV from *Sphingomonas* sp. A1 (GenBank: BAB03319.1) which degrades alginate or its endolytic products into monouronates (4) exolytic alginate lyase, FspAlyFRB from *Falsirhodobacter* sp. *a*lg1 (GenBank: LC081342.1), which is maximally active in temperature range of 25-30 °C, between pH 6–9, (5) endolytic alginate lyase, *Psp*CY24AlyPI from *Pseudoalteromonas* sp. CY24 (GenBank: ACM89454.1), which shows activity at 40 °C and pH 7.0 with enhancement of activity by divalent metal ions, Ca^2+^, Mn^2+^, Na^+^, K^+^ and Fe^3+^, (6) endolytic alginate lyase, *Vsp*AlyVI from *Vibrio* sp. QY101 (GenBank: AAP45155.1), which shows activity towards both polyM and polyG, with optimal conditions of 40 °C and pH 7.5 in the presence of ZnCl_2_, and (7) endolytic alginate lyase, *Ca*Aly from *Cellulophaga algicola* (alginate lyase domain comprising amino acid residues 397 to 634 in Genbank: ADV50193.1, WP_013551659.1) which we have recently demonstrated to be superior to all the above mentioned alginate lyases at inhibiting biofilm formation by *P. aeruginosa* strain MCC 2081 (ATCC 9027, DSM 1128; a strain first isolated from an outer ear infection by C. P. Hegarty, and since used in several studies as a standard strain for its exemplary biofilm forming ability [[6], [7], [8], [9], [10], [11]].

In this study, we have assessed the efficacy of these alginate lyases in inhibiting biofilm formation by a mucoid *P. aeruginosa* strain isolated from a cystic fibrosis patient. Our results indicate that the alginate lyases, *Ca*Aly, *Vsp*AlyVI, *Fsp*AlyFRB, and SA1-IV, indeed inhibit biofilm formation, and are comparable to the glycoside hydrolase, PslG (GenBank: AAG05625.1), a promising candidate for biofilm treatment [12,13]. Our study underscores the necessity of future studies to assess their efficacy in in vivo conditions.

## 2 Materials and methods

***Cloning, expression and purification of alginate lyases and PslG:*** The genes for alginate lyases and PslG used in the study were cloned using codon-optimized gene sequences synthesized by Genscript in pUC57 and subcloned in pET-28a(+) within NcoI and XhoI sites such that they were expressed with a C-terminal 6XHis tag [11].

For *Ca*Aly, only the alginate lyase domain comprising amino acid residues 397 to 634 in Genbank: ADV50193.1, WP_013551659.1 was used for codon-optimization and subsequent cloning and recombinant expression. The expression of all recombinant alginate lyases was induced by the addition of IPTG, and the expressed recombinant proteins were purified from the clarified cell lysate by Ni-NTA affinity chromatography as described [11]. For PslG, *E. coli* BL21 (DE3) cells were transformed with the *pslG*-pET-28a(+). The cultures were induced with 1 mM IPTG (GoldBio) and incubated with shaking at 37 °C for 5 h at 200 rpm. The recombinant protein was purified by column chromatography using Ni-NTA resin (Pierce). Protein purification of all recombinant proteins was as follows. The bacterial cell pellet was disrupted by a probe-type ultrasonicator (Sonics & Materials NC) for 30 min (pulse-10s on and 10s off, amplitude 20%) in lysis buffer (20 mM Tris, 150 mM NaCl, pH 7.5 for all recombinant alginate lyases except SA1-III, for which phosphate buffer, 20 mM with 150 mM NaCl, pH 7.5 was used; 20 mM Tris, 150 mM NaCl, pH 7.5 supplemented with 0.5% N-Lauroylsarcosine for PslG), followed by centrifugation at 16000×*g* for 40 min. The clarified lysate was incubated with Ni-NTA resin (pre-equilibrated with lysis buffer) with end-over-end rotation on a Rotospin (Tarsons) for 2.5 h at 4 °C, and the column was washed extensively prior to elution of the 6XHis tagged protein with imidazole. For PslG, the column was washed with 16 ml of TBS (20 mM Tris, 150 mM NaCl, pH 7.5) containing 30 mM imidazole and the protein was eluted in 8 fractions of 400 μl each with TBS containing 300 mM imidazole. The imidazole eluted protein was extensively dialyzed against TBS (20 mM Tris buffer containing 150 mM NaCl, pH 7.5), the purity and the yield of the protein preparation evaluated by SDS-PAGE and OD_280_ measurement, respectively, and the protein aliquoted and stored at −80 °C until further use.

***Biofilm formation:*** Mucoid *P. aeruginosa* strain 2843 was isolated from sputum of a cystic fibrosis patient. *P. aeruginosa* strain 2081 was obtained from National Centre for Microbial Resource, Pune. For biofilm formation, *P. aeruginosa* strains (2081 and 2843) were cultured in cation-adjusted Mueller Hinton Broth (CAMHB) and statically incubated in a 96-well microtitre plate (200 μl of 1 x 10^7^ cfu per well) at 37 °C for 24 h. The biofilm formed was subsequently fixed by incubating wells with methanol for 15 min. Upon fixing, the biofilm was stained with 0.1% crystal violet as described [11]. In this assay, CAMHB was used as a negative control.

**Determination of minimum inhibitory concentration (MIC) for colistin and tobramycin:** We determined the MIC by broth microdilution method as described in the Clinical and Laboratory Standards Institute (CLSI) guidelines [14]. Dilutions of colistin and tobramycin ranged from 512 to 0.125 μg/ml and were made according to CLSI dilution scheme in cation-adjusted Mueller Hinton Broth. 100 μL of each dilution was aliquoted in the wells of a microtitre plate in triplicate. The *P. aeruginosa* strain 2843 was cultured in CAMHB for 4–6 h and then diluted in such a way that inoculation of 10 μL of this suspension into the antibiotic dilutions would yield the final test concentration of the bacteria to be approximately 5 × 10^5^ CFU/ml. The plate was incubated at 37 °C for 24 h under static conditions, and the absorbance then measured at 600 nm. MIC was calculated by non-linear fitting of the data to sigmoidal equation in Sigma Plot 14.0 software.

***Biofilm inhibition by alginate lyases or PslG:*** Alginate lyase and PslG stocks of 50 μM concentration were prepared for use at a final concentration of 5 μM in the reaction mix of the inhibition assay. For inhibition assays, 20 μl of the alginate lyases was added to the wells of a microtitre plate containing 180 μl of *P. aeruginosa* 2843 culture suspension (total of 1 x 10^7^ cfu per well). Each condition was set up in triplicate. The plate was statically incubated for 24 h at 37 °C. The biofilm formed was subsequently fixed by incubating wells with methanol for 15 min. Upon fixing, the biofilm was stained with 0.1% crystal violet as described [11]. For controls, colistin and tobramycin were added at ~10X MIC to ensure complete inhibition of bacterial growth and thereby biofilm formation.

***Effect of alginate lyase on sputum sample:*** Sputum was provided (with informed consent) by the same cystic fibrosis patient from whom *P. aeruginosa* strain 2843 was isolated. Sputum (25 μl; diluted two-fold in 20 mM Tris buffer) was incubated with alginate lyase (25 μl; 5 μM final concentration) in a UV-compatible 96-well microtitre plate, and absorbance at 235 nm was measured every 60 s in a Synergy H1 plate reader. In parallel, positive control reactions were set up in which alginate lyases (25 μl; 5 μM final concentration) were incubated with sodium alginate (25 μl; 10 mg/ml final concentration) in a UV-compatible 96-well microtitre plate, and the absorbance was measured at 235 nm every 60 sections in a Synergy H1 plate reader, as above, to monitor the formation of unsaturated products upon alginate cleavage by alginate lyases. Negative control reactions had buffer in place of alginate lyase. For assays performed with sputum as well as with sodium alginate (control), reactions were started by the addition of alginate lyase at time zero and monitored in a Synergy H1 plate reader for 120 min. All reactions were set up in duplicate.

***Thin Layer Chromatography:*** The reaction mixtures (10 μl each) of the absorbance assays with sputum were subsequently subjected to thin layer chromatography (TLC) on silica gel 60 F254. Standards were also spotted on the TLC sheet. The solvent system used to develop the thin layer chromatogram was 1-butanol: acetic acid: water:: 3:2:2. Following the TLC run, the sheets were dried and stained with 1 mg/ml 1,3-Dihydroxynaphthalene (Sigma) (in 10% sulfuric acid in 50% ethanol). The TLC sheets were then dried over a hot plate for 10–15 min until bands became visible.

## 3 Results

**Fig. 1:**
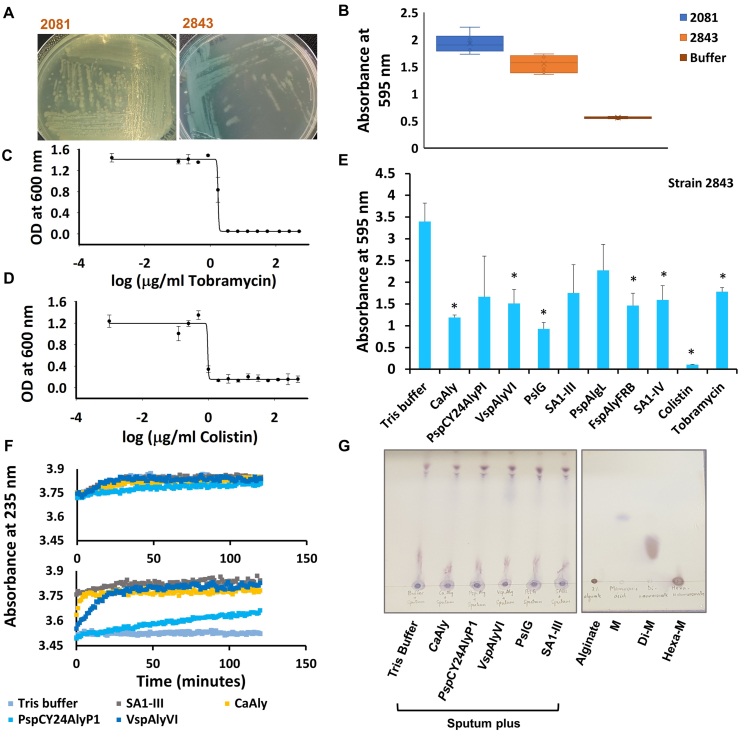
**Effect of alginate lyases on mucoid *P. aeruginosa.* A)** Growth of *P. aeruginosa* strains 2081 and 2843 on agar medium. **B)** Plot showing biofilm formation by mucoid *P. aeruginosa* strain 2843 isolated from a cystic fibrosis patient. Biofilm formation was measured by absorbance of crystal violet staining at 595 nm. Mean values of crystal violet staining of six biological replicates plotted. Error bars signify standard deviation. Results of single experiment, which were representative of two independent experiments with six biological replicates each. **C)** MIC of tobramycin for *P. aeruginosa* strain 2843. Mean values of three biological replicates plotted. Error bars signify standard deviation. Results of a single experiment performed once. **D)** MIC of colistin for *P. aeruginosa* strain 2843. Mean values of three biological replicates plotted. Error bars signify standard deviation. Results of single experiment, which were representative of two independent experiments. **E)** Histogram demonstrating inhibition of biofilm formation of *P. aeruginosa* strain 2843 by alginate lyases, antibiotics (colistin and tobramycin at ~10X MIC to ensure complete bacterial growth inhibition), and glycoside hydrolase, PslG. Mean values of crystal violet staining of three biological replicates plotted. Error bars signify standard deviation. Asterisks show significant inhibition of biofilm formation (p < 0.05 in a two-tailed paired *t*-test). Results of single experiment, which were representative of two independent experiments. **F)** Absorbance based assay to assess alginate lyase activity in sputum (upper panel) and in 2% alginate (lower panel). Results of a single experiment with all reactions set up in duplicate. Results were representative of another experiment performed similarly but containing 20 μg/ml DNase I in sputum. **G)** Thin Layer Chromatogram showing 1,3-Dihydroxynaphthalene stained spots produced from reaction mixtures of absorbance based assay in sputum mentioned in Fig. 1F. Standards are 2% alginate, mannuronic acid, di-mannuronate and hexa-mannuronate. One of the duplicates was spotted for each sample. (For interpretation of the references to colour in this figure legend, the reader is referred to the Web version of this article.)

**Biofilm formation by a *P. aeruginosa* strain isolated from cystic fibrosis sputum:** We isolated an amikacin-resistant, ceftazidime-resistant, imipenem-susceptible, piperacillin-tazobactam-susceptible, levofloxacin-susceptible, netilmicin-susceptible, ciprofloxacin-susceptible and meropenem-intermediately susceptible strain of *P. aeruginosa* strain 2843 from the sputum of a cystic fibrosis patient. *P. aeruginosa* strain 2843 showed mucoid growth on agar plates (Fig. 1A) and biofilm formation on plastic similar to *P. aeruginosa* strain 2081 (Fig. 1B). We found that the minimum inhibitory concentrations (MIC) of tobramycin and colistin for killing planktonic *P. aeruginosa* strain 2843 were 1.73 μg/ml and 0.96 μg/ml, respectively (Fig. 1C, 1D).

**Inhibition of biofilm formation of *P. aeruginosa* 2843 by alginate lyases:** We studied the effect of alginate lyases on biofilm formation by *P. aeruginosa* strain 2843. There was significant crystal violet staining in the wells containing *P. aeruginosa* strain 2843 to which just Tris buffer was added, indicative of good biofilm formation. Further, as expected, we did not observe any crystal violet staining in the wells containing colistin and tobramycin at 10 μg/ml (10XMIC) (p < 0.05, paired T-test). There was no reduction in crystal violet staining in wells containing *P. aeruginosa* to which *Psp*CY24AlyP1 or SA1-III had been added, indicating that biofilm formation was not inhibited by *Psp*CY24AlyP1 and SA1-III (Fig. 1C). However, there was a significant reduction in crystal violet staining in wells containing *P. aeruginosa* to which *Ca*Aly, *Vsp*AlyVI, *Fsp*AlyFRB, SA1-IV or PslG was added, indicating that biofilm formation was significantly inhibited (p < 0.05, paired T-test) by the endotypic alginate lyases, *Ca*Aly, and *Vsp*AlyVI, by the exotypic alginate lyases, *Fsp*AlyFRB and SA1-IV, and by the glycoside hydrolase, PslG (Fig. 1E).

**Effect of alginate lyases on cystic fibrosis sputum:** Considering this positive result, we assessed the effect of these alginate lyases directly on sputum, assuming that alginate would be present in these secretions. Initial incubation of *Ca*Aly with sputum overnight did not result in any visually observable decrease in sputum viscosity in comparison to sputum to which DNase I (final concentration 20 μg/ml) or Tris buffer were added. So we employed absorbance-based and TLC assays for assessing alginate degradation, if any. None of the alginate lyases assayed showed any significant increase in absorbance at 235 nm (Fig. 1F), albeit they were enzymatically active on sodium alginate (Fig. 1F). No spots indicative of alginate degradation could be observed by TLC (Fig. 1G).

## 4 Discussion

The overexpression of the exopolysaccharide, alginate, by mucoid *P. aeruginosa* strains is associated with increased resistance to antibiotics in the cystic fibrosis lung [1,[15], [16], [17]]. However, there are conflicting reports on the action of alginate lyases in inhibiting or eradicating biofilm formation by mucoid *P. aeruginosa* [[3], [4], [5],^18^]. In contrast, the glycoside hydrolase, PslG, which targets the exopolysaccharide, Psl, has been clearly demonstrated to have a significant anti-biofilm effect [12,13,19]. We attempted, in this study, to verify the anti-biofilm action of alginate lyases. We demonstrated that *Pseudomonas* strain 2843 isolated from the sputum of a CF patient demonstrated biofilm formation ability, which was inhibited by *Ca*Aly, *Vsp*AlyVI, *Fsp*AlyFRB and SA1-IV, in a comparable measure to PslG. Our experimental results therefore highlight the potential of certain alginate lyases in combating the biofilms of mucoid *P. aeruginosa* in cystic fibrosis. Albeit we did demonstrate the anti-biofilm effect on the mucoid strain in vitro, a limitation of our study is that we could not demonstrate alginate degradation upon incubation of alginate lyases with sputum. However, it is important to note that we could only assay a single sample of sputum in this study, which might not be indicative of the potential of alginate lyases in sputum. It is also possible that our inability to identify alginate degradation products was due to the inhibition of alginate lyase activity by Zn^2+^ and Ca^2+^ ions present in sputum samples of cystic fibrosis patients (Mrsny et al., 1994), susceptibility of alginate lyases to proteases in the sputum, or presence of only minor amounts of alginate in the collected sputum. We could not determine the amount of alginate in the sputum sample collected but 80–200 μg alginate is expected to be present per ml of sputum in cystic fibrosis patients (Mrsny et al., 1994). Albeit sputum can be collected relatively easily and non-invasively, it is a very complex sample that can be contaminated with saliva and nasal secretions and might not accurately reflect the lung environment. Our study highlights the potential of alginate lyases in combating *P. aeruginosa* biofilms and the necessity of testing the in vivo efficacy of these alginate lyases in future studies perhaps using a large number of sputum samples or other sample types such as bronchoalveolar lavage.

## Author contributions

RTNC conceived the study. MS enrolled patient, supervised the collection of sputum sample, and obtained informed consent. VG isolated the *P. aeruginosa* strain 2843 and performed drug susceptibility testing. SM and SS performed the biochemical experiments with the strain and sputum, respectively. SM wrote the first draft of the manuscript. All authors participated in preparation of the final manuscript.

## Funding

This work was supported by the 10.13039/501100001412Council of Scientific and Industrial Research, Government of India (CSIR-IMTECH Research Council approved project OLP0554). SM and SS acknowledge the 10.13039/501100001407Department of Biotechnology, Government of India and the 10.13039/501100001501University Grants Commission, Government of India for their fellowships.

## Declaration of competing interest

The authors declare that they have no known competing financial interests or personal relationships that could have appeared to influence the work reported in this paper.

## References

[1] Ramsey D.M., Wozniak D.J. (2005). Understanding the control of *Pseudomonas aeruginosa* alginate synthesis and the prospects for management of chronic infections in cystic fibrosis. Mol. Microbiol..

[2] Alipour M., Suntres Z.E., Omri A. (2009). Importance of DNase and alginate lyase for enhancing free and liposome encapsulated aminoglycoside activity against *Pseudomonas aeruginosa*. J. Antimicrob. Chemother..

[3] Alkawash M.A., Soothill J.S., Schiller N.L. (2006). Alginate lyase enhances antibiotic killing of mucoid *Pseudomonas aeruginosa* in biofilms. APMIS.

[4] Lamppa J.W., Griswold K.E. (2013). Alginate lyase exhibits catalysis-independent biofilm dispersion and antibiotic synergy. Antimicrob. Agents Chemother..

[5] Mrsny R.J., Lazazzera B.A., Daugherty A.L., Schiller N.L., Patapoff T.W. (1994). Addition of a bacterial alginate lyase to purulent CF sputum in vitro can result in the disruption of alginate and modification of sputum viscoelasticity. Pulm. Pharmacol..

[6] Mai-Prochnow A., Bradbury M., Murphy A.B. (2015). Draft genome sequence of Pseudomonas aeruginosa ATCC 9027 (DSM 1128), an important rhamnolipid surfactant producer and sterility testing strain. Genome Announc..

[7] Zhou G., Peng H., Wang Y.S., Li C.L., Shen P.F., Huang X.M., Xie X.B., Shi Q.S. (2019). Biological functions of nirS in Pseudomonas aeruginosa ATCC 9027 under aerobic conditions. J. Ind. Microbiol. Biotechnol..

[8] Zhou G., Wang Y.S., Peng H., Shen P.F., Xie X.B., Shi Q.S. (2019). Functional roles of norCB in Pseudomonas aeruginosa ATCC 9027 under aerobic conditions. J. Basic Microbiol..

[9] Jayal A., Johns B.E., Purdy K.J., Maddocks S.E. (2017). Draft genome sequence of Pseudomonas aeruginosa ATCC 9027, originally isolated from an outer ear infection. Genome Announc..

[10] Di Onofrio V., Gesuele R., Maione A., Liguori G., Liguori R., Guida M., Nigro R., Galdiero E. (2019). Prevention of Pseudomonas aeruginosa biofilm formation on soft contact lenses by allium sativum fermented extract (BGE) and cannabinol oil extract (CBD). Antibiotics.

[11] Mahajan S., Ramya T.N.C. (2020). *Cellulophaga algicola* Alginate Lyase Inhibits Biofilm Formation of a Clinical *Pseudomonas aeruginosa* Strain MCC 2081.

[12] Yu S., Su T., Wu H., Liu S., Wang D., Zhao T., Jin Z., Du W., Zhu M.J., Chua S.L., Yang L., Zhu D., Gu L., Ma L.Z. (2015). PslG, a self-produced glycosyl hydrolase, triggers biofilm disassembly by disrupting exopolysaccharide matrix. Cell Res..

[13] Baker P., Whitfield G.B., Hill P.J., Little D.J., Pestrak M.J., Robinson H., Wozniak D.J., Howell P.L. (2015). Characterization of the *Pseudomonas aeruginosa* glycoside hydrolase PslG reveals that its levels are critical for Psl polysaccharide biosynthesis and biofilm formation. J. Biol. Chem..

[14] Jean P., Patel B., Melvin M., Weinstein P., George M., Eliopoulos M., Stephen P., Jenkins G., James P., Lewis S., Brandi Limbago P., Amy M., Mathers J., Tony Mazzulli M., Patel M. Robin, Sandra M., Richter S., Michael Satlin M., Jana M., Swenson M., Maria B., Traczewski M., John M., Turnidge D., Barbara P., Zimmer L. (2015). M07-A10 Methods for Dilution Antimicrobial Susceptibility Tests for Bacteria that Grow Aerobically.

[15] Bayer A.S., Speert D.P., Park S., Tu J., Witt M., Nast C.C., Norman D.C. (1991). Functional role of mucoid exopolysaccharide (alginate) in antibiotic-induced and polymorphonuclear leukocyte-mediated killing of Pseudomonas aeruginosa. Infect. Immun..

[16] Nivens D.E., Ohman D.E., Williams J., Franklin M.J. (2001). Role of alginate and its O acetylation in formation of Pseudomonas aeruginosa microcolonies and biofilms. J. Bacteriol..

[17] Pier G.B., Coleman F., Grout M., Franklin M., Ohman D.E. (2001). Role of alginate O acetylation in resistance of mucoid Pseudomonas aeruginosa to opsonic phagocytosis. Infect. Immun..

[18] Hatch R.A., Schiller N.L. (1998). Alginate lyase promotes diffusion of aminoglycosides through the extracellular polysaccharide of mucoid Pseudomonas aeruginosa. Antimicrob. Agents Chemother..

[19] Ma L., Lu H., Sprinkle A., Parsek M.R., Wozniak D.J. (2007). Pseudomonas aeruginosa Psl is a galactose- and mannose-rich exopolysaccharide. J. Bacteriol..

